# GAM-Based Mooring Alignment for SINS Based on An Improved CEEMD Denoising Method

**DOI:** 10.3390/s19163564

**Published:** 2019-08-15

**Authors:** Hanxiao Rong, Yanbin Gao, Lianwu Guan, Qing Zhang, Fan Zhang, Ningbo Li

**Affiliations:** Collage of Automation, Harbin Engineering University, Harbin 150001, China

**Keywords:** SINS self-alignment, gravitational apparent motion, complementary ensemble empirical mode decomposition, similarity measure, *l*_2_-norm

## Abstract

To solve the self-alignment problem of the Strapdown Inertial Navigation System (SINS), a novel adaptive filter based on Complementary Ensemble Empirical Mode Decomposition (CEEMD) is proposed. The Gravitational Apparent Motion (GAM) is used in the coarse alignment, and the problem of obtaining the attitude matrix between the body frame and the navigation frame is attributed to obtaining the matrix between the initial body frame and the current navigation frame using two gravitational apparent motion vectors at different moments. However, the accuracy and time of this alignment method always suffer from the measurement noise of sensors. Thus, a novel adaptive filter based on CEEMD using an $l_{2}$-norm to calculate the similarity measure between the Probability Density Function (PDF) of each Intrinsic Mode Function (IMF) and the original signal is proposed to denoise the measurements of the accelerometer. Furthermore, the advantage of this filter is verified by comparing with other conventional denoising methods, such as PDF-based EMD (EMD-PDF) and the Finite Impulse Response (FIR) digital low-pass filter method. The results of the simulation and experiments indicate that the proposed method performs better than the conventional methods in both alignment time and alignment accuracy.

## 1. Introduction

The Strapdown Inertial Navigation System (SINS) has been applied in various fields and developed rapidly because of its independence and accuracy [1,2,3,4,5]. The self-alignment, as the basis of SINS, is required to obtain the initial attitude accurately and quickly only by utilizing the measurements from the Inertial Measurement Unit (IMU). Unfortunately, due to the harsh environments and measurement interference, achieving high precision initial alignment within a short time is a great challenge.

Normally, the self-alignment process contains two consecutive phases: coarse alignment and fine alignment [4,6]. The main purpose of coarse alignment is to calculate the rough attitude angles rapidly followed by the Kalman filter-based fine alignment. The precision of coarse alignment determines the accuracy and time of fine alignment. The classical static and quasi-static bases alignment methods can achieve satisfactory results, but they cannot be used for a swaying base due to the disturbance of waves, and so on.

Inspired by the new alignment algorithm of xSea Company, several Gravitational Apparent Motion (GAM)-based coarse alignment methods have been proposed [4,7,8,9]. The alignment problem is converted from determining the attitude matrix between the body frame and the navigation frame to determining the matrix between the body inertial frame and the navigation inertial frame by the GAM-based alignment method. It has been proven that the GAM-based alignment method has the same theoretical alignment accuracy as the conventional methods [7,8]. In practical applications, however, because the accelerometer data are applied to calculate the GAM directly, some non-negligible errors, especially random noise from accelerometers, are brought into the alignment process [8,10]. In response to this problem, Xu J.et al. [6] proposed to adopt velocity vectors by integrating gravitational acceleration to participate in alignment calculation. Nevertheless, in the case of linear velocity interference, the performance of this method will be poor without the reference of external velocity sensors. Chang L. et al. [3] also employed gradient descent optimization to determine the initial attitude matrix according to the characteristics of GAM. The shortcoming of this method is that gradient descent optimization has strict requirements on the objective function and step-size, which limits its wide application in practice. Besides, Liu X. et al. [10] recognized and reconstructed the GAM by analyzing the general expressions of apparent motion. However, the reconstructed system was unable to maintain the complete observability of the whole coarse alignment process. Based on the different frequency characteristics of noise, Xu X. et al. [11] and Sun F. et al. [9] employed the low filter to filter random noises. Meanwhile, Xu X. et al. [12] filtered the high-frequency noises of the measurements with the designed Real-time Wavelet Denoising (RWD). However, due to the instability of external conditions, it is difficult to determine the parameters of the above filters in practical applications. An alternative denoising method was presented by Huang called Empirical Mode Decomposition (EMD) [13,14], which is totally adaptive. To surmount the defect of mode mixing and end effect in conventional EMD, Complementary Ensemble Empirical Mode Decomposition (CEEMD) was proposed in [15]. Then, the Complete Ensemble Empirical Mode Decomposition with Adaptive Noise (CEEMDAN) was proposed as a more effective method [16]. However, the improvement of the filtering accuracy of CEEMDAN is based on longer decomposition time, which makes it restrictive in practical applications. Focusing on the problem of effective IMF selection, Ayenu-Prah et al. [17] and Duan et al. [18] adopted a Correlation-based method (EMD-COR) to determine the effective IMFs. Wang Y. et al. [19] employed EMD-COR to complete the initial alignment of SINS. However, the relevant modes were selected based on prior information, actually. The performances of some methods will be verified later in this paper. To solve this imperfection, a method based on Consecutive Mean Squared Error (CMSE) was proposed in [20]. This method does not need a threshold and can adapt to most conditions, but it will perform poorly because of the local minimum. On the basis of the above studies, Komaty obtained effective IMFs according to the similarity measure between the Probability Density Functions (PDF) of IMFs and the original signal [21]. Yang proposed EMD Interval Thresholding (EMD-IT) based on the probability density function with the order of time complexity comparable to EMD, but having higher accuracy [22]. The limitation of the above two methods is that the mode mixture of EMD has not been fundamentally solved.

Given the problems above, this paper introduces a GAM-based self-alignment method by a novel adaptive filter called effective IMF selection based on CEEMD-$l_{2}PDF$. The main superiority of this method is to select IMFs self-adaptively without any prior information. Measurement signals of the accelerometer with noises are decomposed into IMFs through CEEMD. For the acquisition of relative IMFs, their PDFs are estimated by the kernel density estimator, followed by self-adaptive separation of the main signal and harmful noise. The final reconstructed signal will be applied to calculate GAM in self-alignment. The remainder part of this paper is organized as follows. Section 2 provides the general alignment algorithm based on GAM and the corresponding simulation. Then, the improved denoising method by CEEMD-$l_{2}PDF$ and reconstructed gravitational apparent motion vectors are presented in Section 3. Moreover, simulations, the turntable test, and the ship experiment are carried out to verify the effectiveness of the proposed algorithm in Section 4, whilst the conclusions are given in Section 5.

## 2. Alignment Algorithm Based on GAM and Simulation

### 2.1. An Initial Alignment Method Based on GAM

Due to the complicated marine environment, it is difficult to separate the Earth rate from the measured angular rate badly disturbed by the swaying conditions [7,8,10]. In order to determine the attitude matrix $C_{b}^{n}\left(t\right)$ between the body frame and the navigation frame, it can be decomposed into two parts according to the matrix chain multiplication:

(1) $$C_{b}^{n}\left(t\right)=C_{i_{b0}}^{n}\left(t\right)C_{b}^{i_{b}}\left(t\right)$$

in which *n* and *b* denote the navigation frame and the body frame, respectively; $i_{b0}$ is defined as the inertial frame non-rotating relative to the inertial space, which is fixed with the body frame at time instant t=t(0); $C_{b}^{i_{b0}}\left(t\right)$ is the attitude matrix between the initial body frame and the present body frame, which can be updated by Equation (2).

(2) $${\dot{C}}_{b}^{i_{b0}}\left(t\right)=C_{b}^{i_{b0}}\left(t\right)\left({\tilde{\omega }}_{i_{b0}b}^{b}\times \right)$$

where ${\tilde{\omega }}_{i_{b0}b}^{b}\times$ denotes the angular rate measured by the gyroscope. From the above analysis, it can be concluded that the key point of obtaining $C_{b}^{n}\left(t\right)$ is the calculation of $C_{i_{b0}}^{n}\left(t\right)$ in Equation (1).

**Figure 1:**
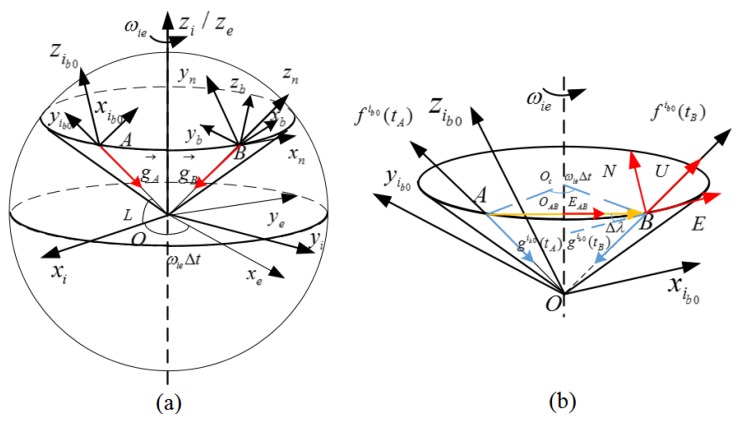
Apparent motion of the gravity vector in the inertial frame: (**a**) The trajectory of the gravity vector; (**b**) Alignment process based on GAM.

According to [6,7], the trajectory of the gravity vector at a fixed point rotating with the Earth is regarded as GAM. As shown in Figure 1a, *A* and *B* represent the same point on the Earth at time $t_{A}$ and current time $t_{B}$ in the inertial frame, and $g_{A}$ and $g_{B}$ are non-collinear gravity vectors of *A* and *B*, respectively. After the rotation of the gravity vector in the Earth cycle, a cone with the geocentric vertex can be formed. In this paper, East–North–Up (ENU) and right-forward-up are selected as frame *n* and frame *b*, respectively. According to Figure 1a, the theoretical measurement of the accelerometer is the projection of the gravity vector in body frame *b* without translational motion of the carrier, and two vectors are in opposite directions as follows:

(3) $$f^{b}\left(t\right)=-g^{b}\left(t\right)$$

where $f^{b}\left(t\right)$ denotes the theoretical measurement of the accelerometer. In practice, the acceleration, ${\tilde{f}}^{b}\left(t\right)$, measured by the accelerometer contains $f^{b}\left(t\right)$ and measurement errors. The projection of ${\tilde{f}}^{b}\left(t\right)$ in the initial inertial frame $i_{b0}$ is as follows:

(4) $${\tilde{f}}^{i_{b0}}\left(t\right)=C_{b}^{i_{b0}}\left(t\right){\tilde{f}}^{b}$$

According to the geometric relationship in Figure 1, Equation (5) can be obtained.

(5) $$E_{AB}=\frac{{\tilde{f}}^{i_{b0}}\left(t_{B}\right)-{\tilde{f}}^{i_{b0}}\left(t_{A}\right)}{∥{\tilde{f}}^{i_{b0}}\left(t_{B}\right)-{\tilde{f}}^{i_{b0}}\left(t_{A}\right)∥}$$

in which $E_{AB}$ is defined as the unit east vector at the middle point $O_{AB}$ between *A* and *B*. In Figure 1b, the navigation frame of point *B* is composed of E, N, and U, which are the east vector, north vector and up vector, respectively. They can be denoted as follows:

(6) $$\begin{array}{c} U\left(t_{B}\right)=\frac{{\tilde{f}}^{i_{b0}}\left(t_{B}\right)}{∥{\tilde{f}}^{i_{b0}}\left(t_{B}\right)∥}\\ E\left(t_{B}\right)=\left[\begin{array}{ccc} \cos\left(\omega_{ie}\left(t_{B}-t_{A}\right)/2\right) & -\sin\left(\omega_{ie}\left(t_{B}-t_{A}\right)/2\right) & 0\\ \sin\left(\omega_{ie}\left(t_{B}-t_{A}\right)/2\right) & \cos\left(\omega_{ie}\left(t_{B}-t_{A}\right)/2\right) & 0\\ 0 & 0 & 1 \end{array}\right]E_{AB}\\ N\left(t_{B}\right)=U\left(t_{B}\right)\times E\left(t_{B}\right) \end{array}$$

where $\omega_{ie}$ is the Earth rate. According to the above vectors, the $C_{i_{b0}}^{n}\left(t_{B}\right)$ can be constructed as follows:

(7) $$C_{i_{b0}}^{n}\left(t_{B}\right)=\left[E\left(t_{B}\right)\;N\left(t_{B}\right)\;U\left(t_{B}\right)\right]$$

According to Equations (1)–(7), the initial alignment for SINS can be accomplished. It has been deduced that the highest theoretical alignment accuracy of this method can be determined as [8]:

(8) $$\phi_{E}=-\frac{\nabla_{N}}{g},\phi_{N}=-\frac{\nabla_{E}}{g},\phi_{U}=-\frac{\varepsilon_{E}}{\omega_{ie}\cdot \cos L}+\frac{\nabla_{E}}{g}\tan L$$

where $E_{E}$ denotes the gyroscope error in the east; $\nabla_{N}$ and $\nabla_{E}$ are the equivalent accelerometer errors in the north and east, respectively.

### 2.2. Simulation

The mooring condition was taken as the simulation environment, and the linear acceleration caused by the surge and sway of the carrier was ignored. The data of acceleration and angular rate were generated by a swaying SINS simulator, and the parameters of the gyroscope and accelerometer are shown in Table 1. In the simulation, the local latitude and the sampling rate respectively were 45.777${}^{\circ }$ and 100 Hz. The initial attitude between the *b*-frame and *n*-frame was set as $\mathrm{pitch}\left(0\right)=10^{\circ }$, $\mathrm{roll}\left(0\right)=0^{\circ }$, and $yaw\left(0\right)=40^{\circ }$, respectively. The swing parameters of the attitude are as follows:

(9) $$\left\{\begin{array}{c} \rho =10^{\circ }+10^{\circ }\sin\left(\frac{2}{7}\pi t\right)\\ \theta =10^{\circ }\sin\left(\frac{2}{5}\pi t\right)\\ \psi =40^{\circ }+5^{\circ }\sin\left(\frac{2}{5}\pi t\right) \end{array}\right.$$

**Figure 2:**
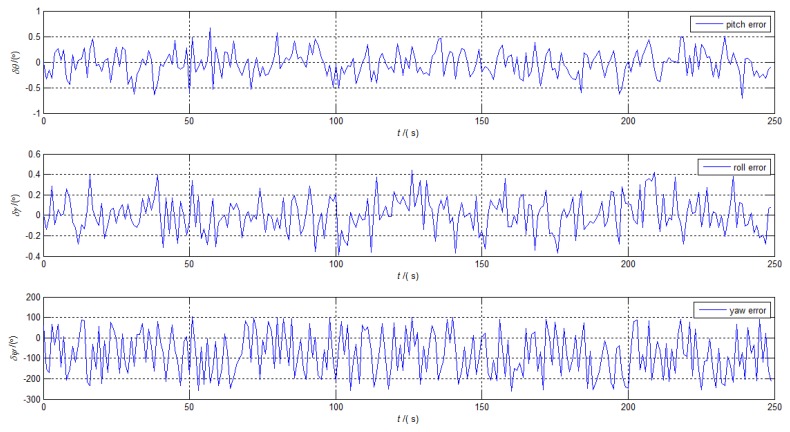
The alignment errors of the Gravitational Apparent Motion (GAM).

The vectors of the current moment and the previous moment were selected as the corresponding GAM vectors in the alignment solution. The update frequency of the alignment solution was 100 Hz. Considering the requirement of rapidity in coarse alignment, the alignment process took 250 s. The alignment errors of the above method are shown in Figure 2, and the corresponding Standard Deviation (STD) and means of the alignment errors in last the 10 s are shown in Table 2.

According to Equation (8) and Table 1, the errors of the above alignment method caused by IMU biases were about $\left(-0.006^{\circ },0.006^{\circ },-0.06^{\circ }\right)$. The curves in Figure 2 indicate that the horizontal errors can approach the theoretical accuracy within 250 s, while the azimuth error cannot satisfy this. As shown in Figure 2, the curve of the yaw error remains oscillatory, which means that the error will not converge with the increasing of the alignment time. There are two main reasons for the alignment failure when analyzing the alignment process of the above methods. One reason is the GAM vectors become approximate collinear due to the existence of noise. The other reason is the random noise makes it difficult to obtain real GAM from the measured data.

## 3. Improved Method Based on CEEMD

To solve the problems in Section 2, the works in [11,12] adopted the low-pass digital filter method and the RWD method, respectively. Considering the signal characteristics of the IMU, an improved CEEMD-based method is proposed in this paper. The significant differences of the proposed method compared to the filtering methods mentioned in [9,11] are that it is adaptive and it does not require any prior information.

### 3.1. A Brief Review of the CEEMD Method

The EMD-based denoising method can decompose the original signal into several IMFs and a residue adaptively based on the intrinsic characteristics of the signal. Therefore, the EMD-based denoising method has been adopted as an effective method for dealing with the nonlinear and the non-stationary signals [21]. As EMD proved to be, it still leaves the problems of mode mixing and end effect unresolved. To solve the phenomenon of mode mixing and the end effect in the original EMD, EEMD was proposed in [14]. The main strategy of EEMD is that the intrinsic local oscillations can be filtered adaptively to proper scales via the natural filter bank of EMD by adding uniformly-distributed white noise. Practically, however, the residue noise caused by the the added white noise in the signal reconstruction cannot be eliminated by the limited number of ensembles. In other words, the added noise would inevitably bring new errors to the IMFs derived from EEMD. CEEMD is an optimization method of EEMD, which can overcome the drawbacks mentioned above by using each noise in pairs with plus and minus signs. Therefore, the signal composed of the original signal and added white noise can be expressed as:

(10) $$\left[\begin{array}{c} y_{1}\\ y_{2} \end{array}\right]=\left[\begin{array}{cc} 1 & 1\\ 1 & -1 \end{array}\right]\left[\begin{array}{c} x\\ w \end{array}\right]$$

in which $y_{1}$ and $y_{2}$ denote the sum of the original data with positive noise and negative noise, respectively; *x* is the original data; *w* is the added white noise. For given original data x(t), the procedure of CEEMD can be described as follows:

1. Generate reconstructed *y* according to Equation (10).
2. Decompose completely $y_{1}$ and $y_{2}$ by EMD, obtaining $IMF^{i}\left(+\right)$ and $IMF^{i}\left(-\right)$(i=1,…,I) derived from $y_{1}$ and $y_{2}$, respectively, where *i* is the number of IMFs.
3. Compute the $i^{\mathrm{th}}$ mode of x(t) by averaging the corresponding modes: $IMF^{i}=\left(IMF^{i}\left(+\right)+IMF^{i}\left(-\right)\right)/2$.
4. The original signal is eventually decomposed into multiple IMFs and residual signal $r_{I}$ by CEEMD: $x\left(t\right)=\sum_{i=1}^{I}IMF^{i}+r_{I}$.

This scheme has an obvious suppression effect on noise in the reconstructed signal, which will be verified later. Similar to EMD, CEEMD is a data-driven adaptive decomposition approach, which is a sifting process based on the local time scale. In order to ensure that each IMF has physical meaning, the fixed sifting number in this paper is set to 12 according to [23].

### 3.2. Improved CEEMD Denoising Method Based on the $l_{2}$-Norm Measure between the PDFs

For a noiseless signal z(t) contaminated by an additive noise n(t):

(11) x(t)=z(t)+n(t)

The purpose of CEEMD as a filter is essentially obtaining a reconstructed and denoised signal $\tilde{x}\left(t\right)$ based on relevant modes. The selection of the effective mode is an open question, which has a decisive influence on the denoising effect of the original signal. The relevant modes can be selected by a given standard that differentiates the modes carrying potential information relevant to the main structures of the original signal. The reconstructed and denoised signal is given by:

(12) $$\tilde{x}\left(t\right)=\sum_{i=kth}^{I}IMF^{i}+r_{I}$$

where $k^{\mathrm{th}}$ denotes the order of partial reconstruction. One method to determine $k^{\mathrm{th}}$ is to calculate the correlation coefficient between the residual of each mode and the original signal. The order of the mode removed from the original signal is defined as:

(13) $$l=k_{th}-1$$

The residual of each mode can be expressed as:

(14) $$r_{l}\left(t\right)=x\left(t\right)-\sum_{i=1}^{l}IMF^{i}$$

**Figure 3:**
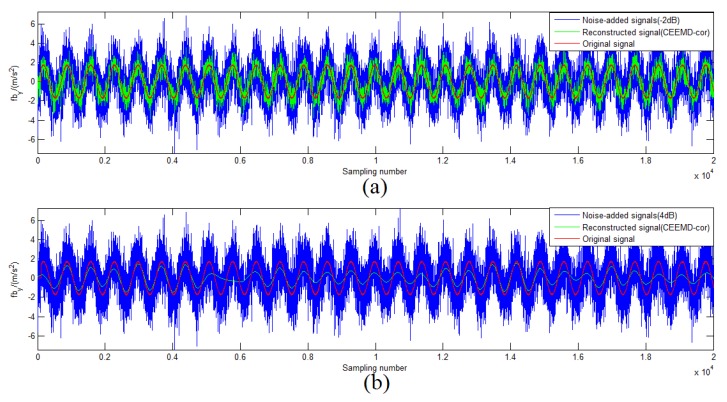
Reconstructed signals for the different noise signals: (**a**) −2-dB SNR noise signals; (**b**) 4-dB SNR noise signals.

The correlation coefficient between x(t) and $r_{l}\left(t\right)$ is as follows:

(15) $$\rho \left(l\right)=\sum_{t=1}^{N}x\left(t\right)r_{l}\left(t\right)/\left(\sqrt{\sum_{t=1}^{N}x^{2}\left(t\right)}\sqrt{\sum_{t=1}^{N}r_{l}^{2}\left(t\right)}\right)$$

where *N* is the length of data. In order to determine $k_{th}$, a threshold *M* should be determined based on the prior information. According to the analysis in [17], Equation (16) can be obtained.

(16) $$u_{th}=\frac{\max(\rho (i))}{10\times \max(\rho (i))-3}$$

where ρ(i) denotes the correlation coefficient between each $IMF^{i}$ and x(t); $u_{th}$ represents the threshold of the uncorrelated IMFs. In this section, the threshold M is determined by $u_{th}$ is 0.75. The $k_{th}$ can be obtained by Equation (17).

(17) $$k_{th}=arg\underset{1\leq l\leq I}{first}\left\{\rho \left(l\right)\leq M\right\}$$

in which “first” means the first value less than *M* in set {ρ(l)}. To analyze the performance of the CEEMD denoising method based on the Correlation coefficient (CEEMD-COR), 50-times Monte Carlo (MC) simulations were conducted in different Signal-to-Noise Ratio (SNR) signals. Figure 3 shows some results of CEEMD-COR denoising with −2 dB and 4 dB SNR noise signals. It can be seen that this method is sensitive to some noisy signals with different SNR.

Inspired by [21], the selection of $k_{th}$ can be tackled by the similarity measure between the PDF of the original signal and each IMF instead of the correlation coefficient. On the basis of Bayesian interpretation, a PDF contains complete information about the signals of interest, rather than merely the oscillation amplitude. The characteristics of PDF make it possible to identify the IMFs dominantly carrying the features of x(t) by a PDF similarity measure. The information-theoretic measures and the metrics-based measures are the two main categories of the similarity measure. It has been proven that geometric similarity measurement based on the $l_{2}$-norm has the best effect in [22].

For given original data x(t) and the result of decomposition by CEEMD $\left(IMF^{1},\ldots ,IMF^{I}\right)$, the $l_{2}$-norm is defined by:

(18) $${∥p^{i}-P∥}_{2}={\left(\int_{-\infty }^{+\infty }{\left(p^{i}\left(z\right)-P\left(z\right)\right)}^{2}dz\right)}^{2}$$

where $p^{i}$ and *P* are the PDFs of $IMF^{i}$ and x(t) obtained by the kernel density estimator. The similarity measure between $p^{i}$ and *P* by the $l_{2}$-norm can be expressed as:

(19) $$D\left(i\right)=\underset{1\leq i\leq I}{dist}\left[p^{i},P\right]$$

where “dist” stands for the distance between two PDFs according to Equation (18). The order of partial reconstruction, $k_{th}$, can be described as follows:

(20) $$k_{th}=arg\underset{1\leq i\leq I}{first}\left\{D\left(i\right)<D\left(i-1\right)\right\}$$

in which “first” means the first order that the distance starts to be smaller than that of the previous order.

**Figure 4:**
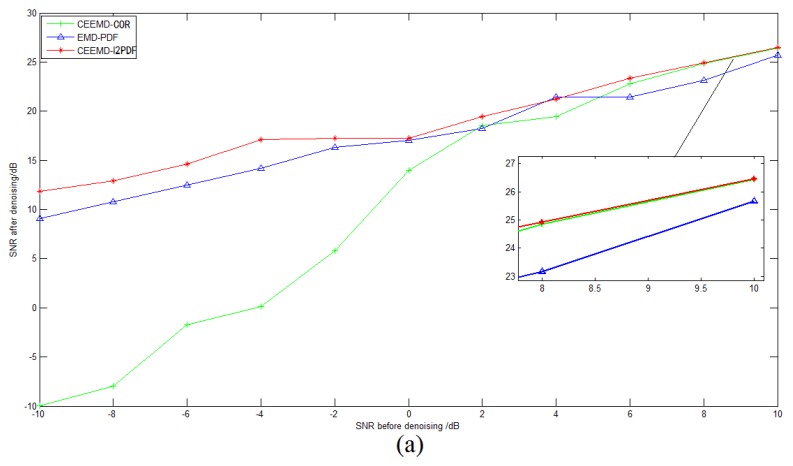

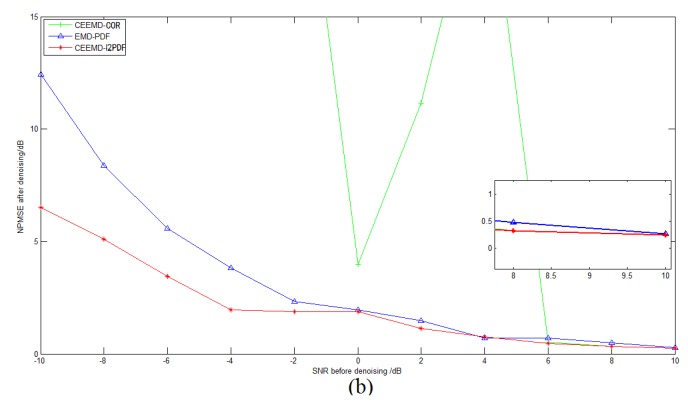


**Figure 5:**
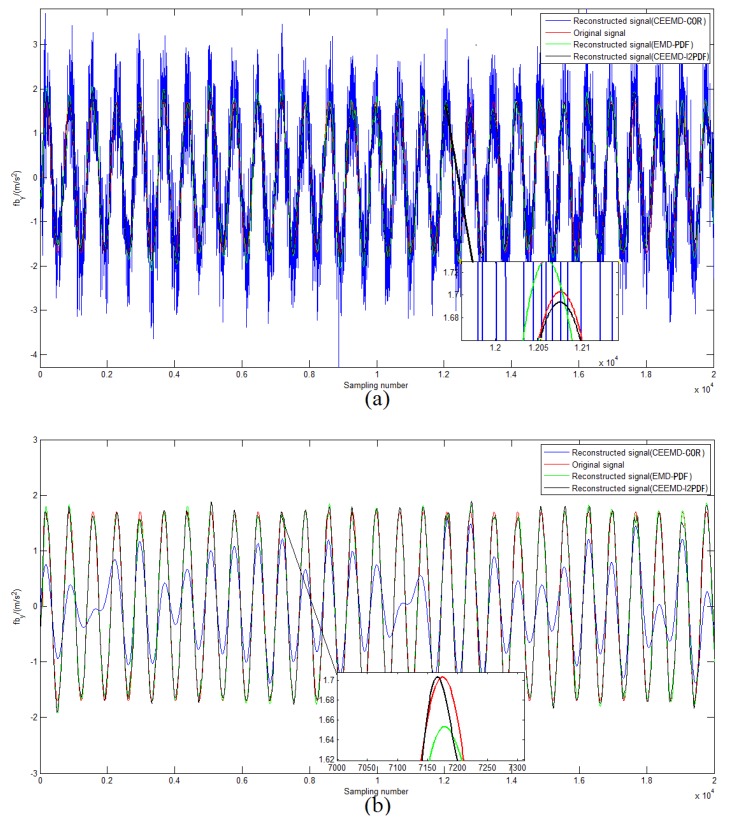
Reconstructed signals by different methods: (**a**) −2-dB SNR noise signals; (**b**) 4-dB SNR noise signals.

To compare the performances of CEEMD-COR, the conventional EMD-PDF, and the proposed CEEMD-$l_{2}PDF$-based method, an MC simulation was performed with the same simulation parameters. The SNR and the Normalized Percent Mean Square Error (NPMSE) were adopted as the measurements of the noise-reducing efficiency [23]. Figure 4a,b shows the comparison of SNR and NPMSE after denoising, respectively. As shown in Figure 4, EMD-PDF and CEEMD-$l_{2}PDF$ performed similarly, but CEEMD-$l_{2}PDF$ showed a better denoising effect than the other two methods with various noise signals. For 8-dB SNR and 10-dB SNR noise signals, the gain of CEEMD-COR was almost equal to that of CEEMD-$l_{2}PDF$, while CEEMD-COR behaved markedly worse than EMD-PDF and CEEMD-$l_{2}PDF$ at other SNR levels. Figure 5 intuitively shows the denoising results of these methods on −2-dB and 4-dB SNR signals in the simulations, respectively. In Figure 4 and Figure 5, the same conclusions can be drawn that CEEMD-$l_{2}PDF$ shows the best robustness and superiority over CEEMD-COR and EMD-PDF on the reconstruction error and variance for signals measured by IMU.

## 4. Simulation and Experiment Results

**Figure 6:**
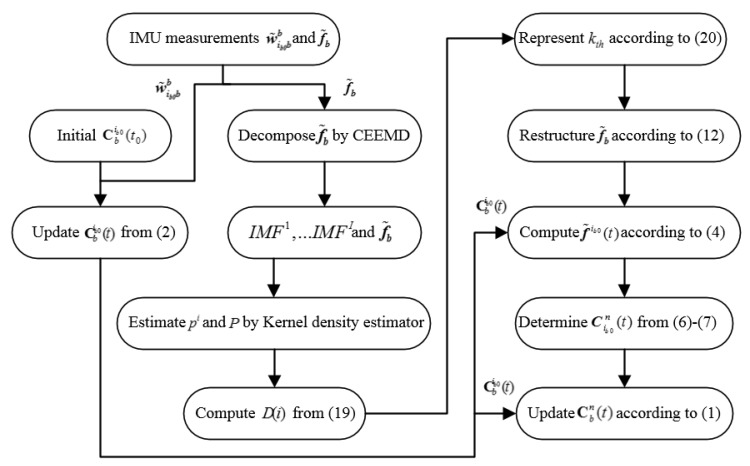
Process of the proposed algorithm.

To evaluate the performances of the proposed SINS self-alignment based on CEEMD-$l_{2}PDF$, the simulation and experiments on the swaying condition were carried out with the comparisons between EMD-PDF, the method of the Finite Impulse Response (FIR) digital low-pass filter proposed in [10], the method of alignment based on the velocity vector, and the proposed method. The procedure of the proposed algorithm can be summarized as shown i Figure 6.

### 4.1. Simulation Results

**Figure 7:**
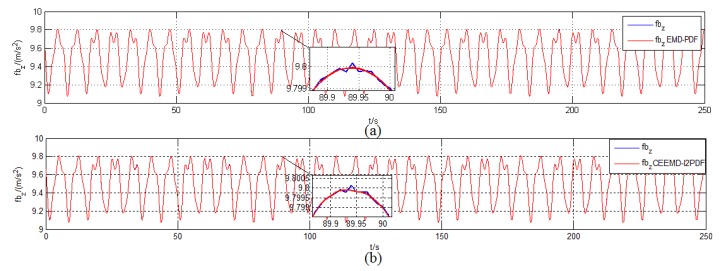
Denoising results of ${\tilde{f}}_{z}^{b}$ in the simulation. (**a**) EMD-PDF method; (**b**) CEEMD-$l_{2}PDF$ method.

**Figure 8:**
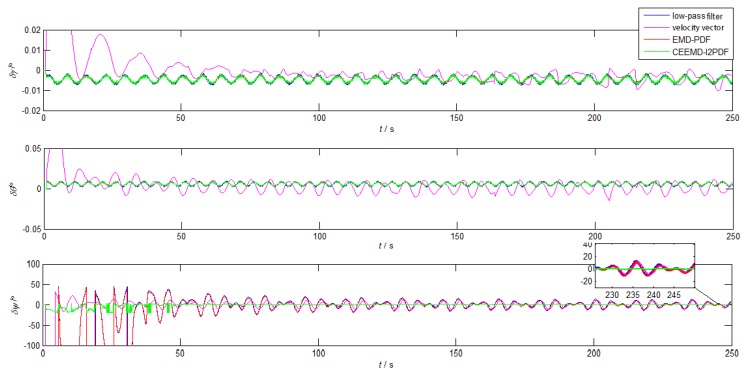
The alignment errors of low-pass, velocity vector, EMD-PDF, and CEEMD-$l_{2}PDF$ in simulation.

The initial conditions and simulation environment were the same as those described in Section 2.2. To compare the filtering effect of EMD-PDF and CEEMD-$l_{2}PDF$, the denoising results of ${\tilde{f}}_{z}^{b}$ for IMU measurement are plotted in Figure 7. The corresponding alignment errors are shown in Figure 8. The mean and Standard Deviation (STD) of alignment errors were adopted to evaluate the alignment effect during the final 10 s.

Figure 7 shows that EMD-PDF and CEEMD-$l_{2}PDF$ had a similar filtering effect when denoising the same signal. However, the slight fluctuation of the original signal can be reflected in the reconstructed signal by CEEMD-$l_{2}PDF$, which was not revealed in EMD-PDF.

The curves in Figure 8 show that the horizontal attitude errors of all methods can achieve the theoretical accuracy within 250 s, while only the yaw errors of CEEMD-$l_{2}PDF$ reached the theoretical accuracy. The horizontal attitude errors of CEEMD-$l_{2}PDF$, EMD-PDF, and the low-pass filter method had similar trends. In addition, CEEMD-$l_{2}PDF$ showed superiority over other methods, especially in convergence time. The alignment error of CEEMD-$l_{2}PDF$ converged to less than 2${}^{\circ }$ in about 46 s, while other methods reached 2${}^{\circ }$ in roughly 230 s. Compared with EMD-PDF, the yaw error based on CEEMD-$l_{2}PDF$ is reduced by 80.7% in Table 3, which greatly improved the accuracy of coarse alignment. According to Figure 8 and Table 3, it can be concluded that the coarse alignment under the swinging condition can be realized by the improved CEEMD-$l_{2}PDF$ denoising method.

### 4.2. Turntable Test Result

**Figure 9:**
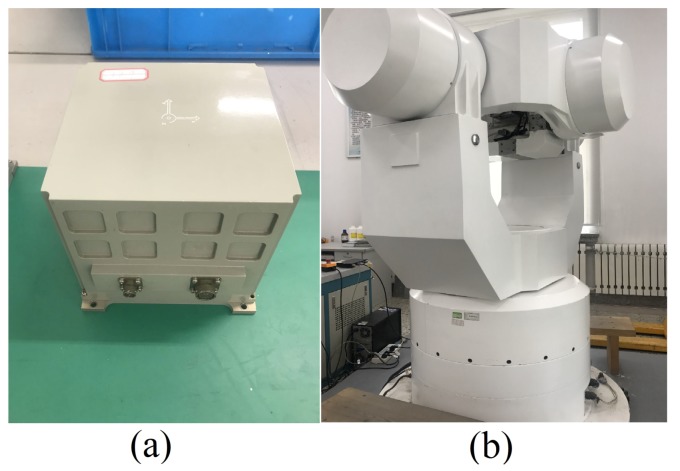
Experimental conditions of the turntable. (**a**) Self-developed SINS. (**b**) Three-axis turntable.

The swaying experiment of the Fiber Optic Gyroscope (FOG)-based SINS on a tri-axial turntable is shown in Figure 9. Table 4 shows the parameters of the self-developed high precision FOG-based SINS. The local latitude was 45.777${}^{\circ }$.

**Figure 10:**
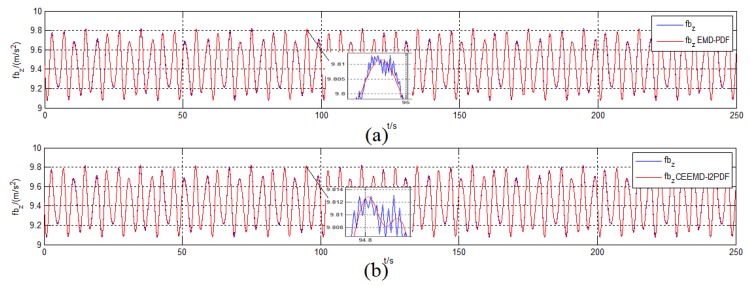
Denoising results of ${\tilde{f}}_{z}^{b}$ in the turntable test. (**a**) EMD-PDF method; (**b**) CEEMD-$l_{2}PDF$ method.

**Figure 11:**
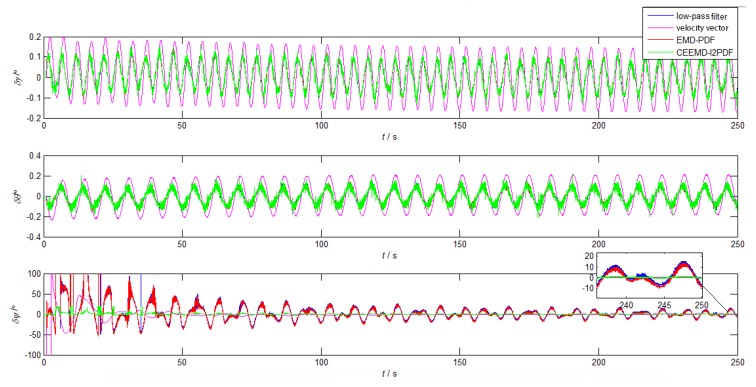
The alignment errors of low-pass, velocity vector, EMD-PDF, and CEEMD-$l_{2}PDF$ in the simulation.

To obtain the reference attitude, we firstly adopted the initial alignment of SINS on the static turntable for 30 min. After that, the turntable began to swing, followed by 250-s coarse alignment. The attitude solution of SINS was regarded as an approximate reference attitude in the turntable test. The bias error of IMU in alignment error was eliminated because of the output of the SINS, which improved the alignment accuracy by the denoising method obviously. Figure 10 and Figure 11 and Table 5 illustrate the results of denoising for ${\tilde{f}}_{z}^{b}$ and corresponding alignment errors in the turntable test, respectively.

Similar to the denoising results in simulation, Figure 10 shows a more significant filtering difference between CEEMD-$l_{2}PDF$ and EMD-PDF. The signal filtered by CEEMD-$l_{2}PDF$ reproduced two peaks of the original signal in about 25 s, but EMD-PDF could not implement it. This indicates that the proposed method can retain more perfect features of the original signal compared with EMD-PDF.

Since the yaw error was one order of magnitude larger than the horizontal error during the coarse alignment, the yaw error was mainly analyzed afterwards. As shown in Figure 11, GAM-based alignment methods based on filtering have advantages in the horizontal alignment, which is consistent with the results of the simulation. However, the method based on the velocity vector showed a smoother and faster convergence than EMD-PDF and low-pass filter method in the azimuth alignment. It took 46 s for the yaw error of the velocity-based method to converge to 3${}^{\circ }$, which is close to that of CEEMD-$l_{2}PDF$ (46 s). From Table 5, it can be seen that the yaw error of the CEEMD-$l_{2}PDF$ method was reduced by 48.4% with respect to the velocity-based method. In addition, because the turntable center did not completely coincide with the SINS center, the existence of the lever arm would cause errors in the alignment results, such as the roll error caused by the acceleration of the lever arm effect in Table 5. Actually, the lever arm error will be compensated by related algorithms. Figure 11 and Table 5 indicate that the CEEMD-$l_{2}PDF$ denoising method was helpful to improve the alignment errors in this turntable test.

### 4.3. Ship Mooring Experiment

**Figure 12:**
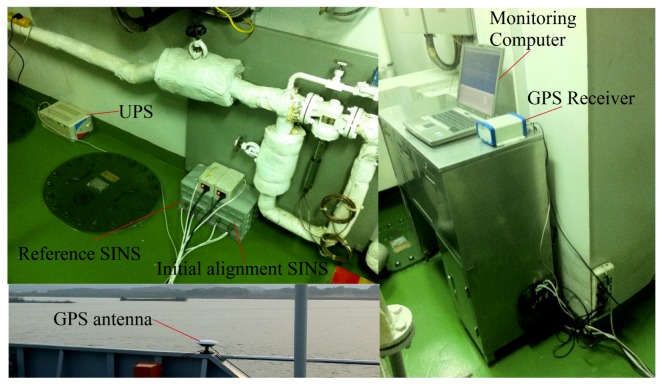
Ship mooring experiment.

**Figure 13:**
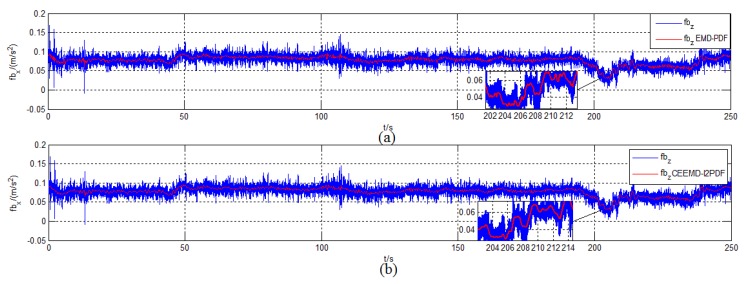
Denoising results of ${\tilde{f}}_{x}^{b}$ in the ship mooring experiment. (**a**) EMD-PDF method; (**b**) CEEMD-$l_{2}PDF$ method.

**Figure 14:**
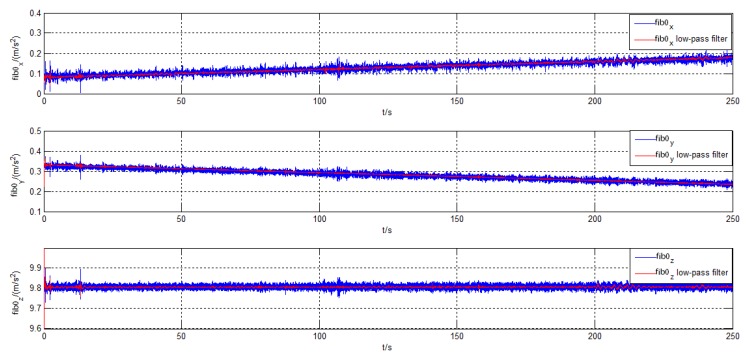
Denoising results of ${\tilde{f}}^{i_{b0}}$ in the ship mooring experiment.

**Figure 15:**
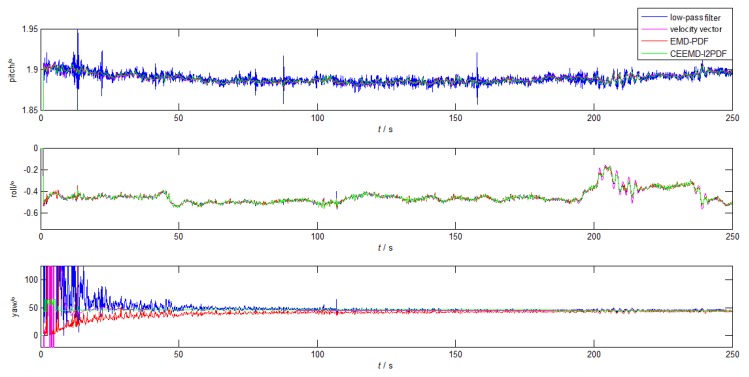
The alignment results in the ship mooring experiment.

**Figure 16:**
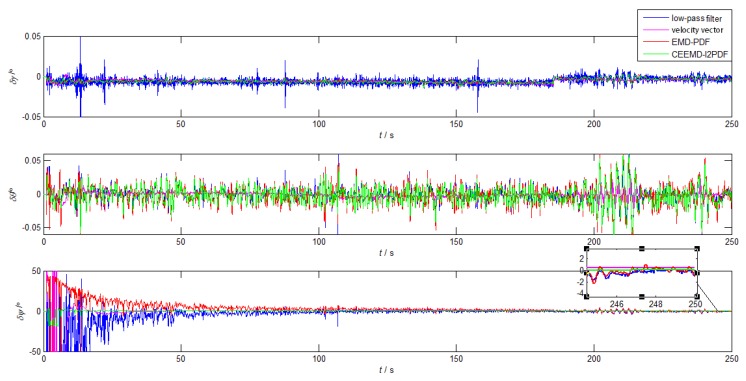
The alignment errors in the ship mooring experiment.

The ship mooring experiment was carried out in South China Sea with two self-developed SINS used in the turntable test. As shown in Figure 12, the GPS/SINS integrated navigation system was used as the attitude reference. The installation error was corrected through the inertial navigation mode. Because of the obvious fluctuation of ${\tilde{f}}_{x}^{b}$ measured by IMU, Figure 13 and Figure 14 illustrate the performances of the CEEMD-based denoising methods for ${\tilde{f}}_{x}^{b}$ and the low-pass filter method for ${\tilde{f}}^{i_{b0}}$. The coarse alignment results and corresponding errors of different methods are presented in Figure 15 and Figure 16, respectively. The means and STDs of the alignment errors during the final 10 s are given in Table 6.

Figure 13 shows that EMD-PDF method and CEEMD-$l_{2}PDF$ method can still effectively eliminate most of the noise in practical applications. Moreover, the signals filtered by the two methods still had different features, which will directly influence the performance of coarse alignment.

In Figure 15, the velocity-based method had a smoother alignment process. However, when the ship was disturbed by wind or waves, the roll of the velocity-based method became more volatile than those of the other three methods.

It can be noticed that the alignment accuracy of the denoising methods in Figure 15 was greatly improved when compared with that in the turntable test, because the amplitude and frequency of the attitude fluctuation were much smaller than those of turntable test, which were mainly caused by the irregular interferences from waves. Because of the linear velocity caused by the ship’s surge and sway, the velocity-based method was inferior to the other methods. In Figure 15 and Table 6, the yaw error of CEEMD-$l_{2}PDF$ showed slightly smaller alignment errors and smaller variance than other methods. Compared with EMD-PDF, the mean and STD of CEEMD-$l_{2}PDF$ were improved by 41.3% and 91.5%, respectively.

Figure 13 and Figure 14 show that the whole coarse alignment can be divided into two stages: the stable alignment phase with little wave interference in the first 190 s and the interfered phase with significant wave-induced interference in the last 60 s. During the stable alignment, the yaw errors of low-pass filter method, velocity-based method, EMD-PDF, and CEEMD-$l_{2}PDF$ reached 2${}^{\circ }$ at 178 s, 27 s, 156 s, and 11 s, respectively. In the subsequent interfered alignment, the maximum fluctuation amplitudes of the yaw errors of these methods caused by wave disturbance were 3.849${}^{\circ }$, 0.760${}^{\circ }$, 2.411${}^{\circ }$, and 0.253${}^{\circ }$. After that, they converged to 0.348${}^{\circ }$, 0.649${}^{\circ }$, 0.227${}^{\circ }$, and 0.027${}^{\circ }$ in 10 s, respectively. CEEMD-$l_{2}PDF$ had strong robustness to interference, while the EMD-PDF and low-pass methods showed obvious fluctuations. Thus, the improved GAM-based alignment algorithm using CEEMD-$l_{2}PDF$ would be more convenient for practical applications.

## 5. Conclusions

Aimed at the problem caused by noise influence in the conventional GAM-based alignment method, a novel denoising method combining the $l_{2}$-norm with the similarity measure of the PDF based on CEEMD was proposed in this paper. Unlike previous methods, it filters the signals based on the intrinsic characteristics of the data by CEEMD. Moreover, the proposed CEEMD-$l_{2}PDF$ method can overcome the distortion of reconstructed signal caused by mode mixing and the end effect. In addition, it adaptively determined the effective IMFs using the similarity measurement between each IMF and the original signal calculated by the $l_{2}$-norm without any prior information. The simulation and turntable test demonstrated the superiority of the proposed method in alignment time and accuracy. Furthermore, the ship mooring experiment indicated that the proposed method possessed better robustness and adaptability in the case of disturbance. Due to the computational complexity of the CEEMD algorithm, the application of the proposed method to real-time data processing needs further study. Although the proposed method had great improvement in alignment accuracy and anti-interference ability, its application in real-time data processing still needs further research. In addition, how to improve CEEMD-$l_{2}PDF$ to reduce the computational complexity brought by the CEEMD algorithm will be the focus of the follow-up work.

## Figures and Tables

**Table 1:** Parameters of the gyroscope and accelerometer.

Parameter Item	Parameter Values
Gyro constant bias (${}^{\circ }$/h)	0.01
Gyro random bias (white noise) (${}^{\circ }/\sqrt{h}$)	0.001
Accelerometer bias (μg)	100
Accelerometer random bias (white noise) (μg)	10

**Table 2:** Means and STDs of the alignment errors from 240 s–250 s.

Parameter	Pitch Error ${(}^{\circ })$	Roll Error ${(}^{\circ })$	Yaw Error ${(}^{\circ })$
Mean	−0.0070	0.0035	−89.6043
STD	0.2609	0.1665	137.3078

**Table 3:** Means and STDs of the alignment errors in the simulation.

Methods	Parameter	Pitch Error $\left({}^{\circ }\right)$	Roll Error $\left({}^{\circ }\right)$	Yaw Error $\left({}^{\circ }\right)$
low-pass	Mean	−0.0012	0.0014	−0.3580
	STD	0.0021	0.0027	1.9790
velocity vector	Mean	−0.0045	0.0011	−0.2450
	STD	0.0029	0.0059	1.3584
EMD-PDF	Mean	−0.0012	0.0014	−0.1457
	STD	0.0021	0.0026	1.8911
CEEMD-$l_{2}PDF$	Mean	−0.0012	0.0015	−0.0277
	STD	0.0021	0.0026	0.1231

**Table 4:** The parameters of self-developed SINS.

Parameter Item	Initial Alignment Mode	Navigation Mode
Horizontal attitude	<0.02${}^{\circ }$ (1σ)	<0.01${}^{\circ }$/h (1σ)
	<0.05${}^{\circ }$ (max)	
Yaw	<0.06${}^{\circ }\sec\Phi$ (1σ)	<0.01${}^{\circ }$/h (1σ)
	<0.10${}^{\circ }\sec\Phi$ (max)	

**Table 5:** Means and STDs of the alignment errors in the turntable test.

Methods	Parameter	Pitch Error $\left({}^{\circ }\right)$	Roll Error $\left({}^{\circ }\right)$	Yaw Error $\left({}^{\circ }\right)$
low-pass	Mean	−0.0003	−0.0028	0.3931
	STD	0.0296	0.0327	3.0755
velocity vector	Mean	−0.0136	−0.0069	0.0462
	STD	0.1848	0.2165	0.0994
EMD-PDF	Mean	−0.0002	−0.0028	0.0898
	STD	0.0297	0.0323	3.0110
CEEMD-$l_{2}PDF$	Mean	−0.0004	−0.0030	0.0238
	STD	0.0305	0.0332	0.1847

**Table 6:** Means and STDs of alignment errors in the turntable test.

Methods	Parameter	Pitch Error $\left({}^{\circ }\right)$	Roll Error $\left({}^{\circ }\right)$	Yaw Error $\left({}^{\circ }\right)$
low-pass	Mean	−0.0008	0.0004	−0.0878
	STD	0.0019	0.0061	0.3396
velocity vector	Mean	−0.0039	0.0046	−0.4737
	STD	0.0004	0.0024	0.0253
EMD-PDF	Mean	−0.0007	0.0009	−0.0242
	STD	0.0015	0.0076	0.3665
CEEMD-$l_{2}PDF$	Mean	−0.0007	0.0009	−0.0142
	STD	0.0015	0.0142	0.0310
